# Supplementary material for: Preclinical Evaluation of TB/FLU-04L—An Intranasal Influenza Vector-Based Boost Vaccine against Tuberculosis
Source: Int J Mol Sci. 2023 Apr 18;24(8):7439. doi: 10.3390/ijms24087439 (PMC10138401; doi:10.3390/ijms24087439)
Supplement: Supplementary file 1 [file ijms-24-07439-s001.zip › ijms-2349359-supplementary.pdf]

International Journal of
Molecular Sciences

Supplementary

Table S1. Experimental monkeys' body temperature

Animal №	Day post-immunization					
	**0**	**2**	**4**	**8**	**20**	**42**
36112	38.2	39.2	38.7	38.3	39.5	38.9
37410	38.3	39.0	38.7	38.6	38.7	38.3
36955	37.2	38.0	38.0	37.6	37.4	37.8
38544	37.2	39.0	39.3	38.4	39.1	39.5
36580	37.9	38.4	38.2	37.5	38.1	37.4
37439	38.5	38.8	38.6	38.2	38.1	38.3
38649	38.7	38.2	39.0	38.2	38.7	37.5
**Mean±SD**	**38.0±0.6**	**38.7±0.6**	**38.6±0.4**	**38.1±0.4**	**38.5±0.7**	**38.2±0.8**

Table S2. Experimental monkeys' body weight

Animal №	Day post-immunization					
	**0**	**2**	**4**	**8**	**20**	**42**
36112	5.60	5.75	5.60	5.55	5.70	5.65
37410	4.65	4.75	4.70	4.50	4.50	4.40
36955	3.10	3.35	3.30	3.30	3.20	3.40
38544	3.50	3.85	3.75	3.60	3.60	3.70
36580	5.00	5.45	5.30	5.25	5.10	5.50
37439	3.10	3.25	3.15	3.15	3.15	3.05
38649	2.55	2.56	2.55	2.50	2.50	2.45
**Mean±SD**	**3.93±1.15**	**4.14±1.20**	**4.05±1.16**	**3.98±1.14**	**3.96±1.16**	**4.02±1.22**

Seven adult male macaques *Macaca fascicularis* were immunized with TB/FLU-04 (7.5 $log_{10}TCID_{50}$/animal) twice, spaced three weeks apart. Macaques were monitored daily during the whole study period. Temperature measurement and body weighing were performed on the indicated dates. *Macaca fascicularis* physiological temperature range is 36.5-39.5ºC

Table S3. Experiment design safety evaluation in mice (Fig 2)

		Day 0		Day 3	Day 5
Group	Number of animals	Immunization		Euthanasia	
TB/FLU-04L	12	TB/FLU-04L 0.01 ml 6.0 log_{10} $TCID_{50}$/animal i.n	12/12	6/12	6/12
PR8	10	A/PR/8/34 0.01 ml 5.0 log_{10} $TCID_{50}$/animal i.n	10/10	5/10	5/10

Groups of 12 or 10 7-8 weeks old female C57bl/6 mice were inoculated with 0.01 ml of either TB/FLU-04L in a dose 6.0 log_{10} $TCID_{50}$/animal or A/PR/8/34 in a dose 5.0 log_{10} $TCID_{50}$/animal under slight ether anesthesia. Viral loads in 10% suspensions of nasal turbines and lungs were determined on days 3 and 5 post-inoculation in Vero cells.

Table S4. Experimental design: immunogenicity evaluation in mice (Fig 3)

		Day 0		Day 21
Group	Number of animals	Immunization		Euthanasia
TB/FLU-04L	6	TB/FLU-04L 0.01 ml 6.0 log$_{10}$ TCID$_{50}$/animal i/n	6/6	6/6
Mock	6	DPBS 0.01 ml i.n	6/6	6/6

Groups of six 7-8 weeks old female C57bl/6 mice were inoculated with 0.01 ml of DPBS (Mock) or TB/FLU-04L in a dose of 6.0 log$_{10}$ TCID$_{50}$/animal under slight ether anesthesia. Three weeks after the vaccination, single-cell suspensions prepared from the lungs and spleens of vaccinated mice were stimulated by the ESAT-6 and Ag85A recombinant proteins *in vitro*. The frequencies of IFN-γ-, TNF-α-, and IL-2-producing CD4$^+$ and CD8+ T$_{em}$ cells were measured by flow cytometry (ICS). Background staining from cells stimulated with medium alone has been subtracted. The data were considered statistically significant when $p<0.05$ in the Mann-Whitney test. Additionally, serums were collected for antibody response determination in HIA.

Table S5. Experimental design: immunogenicity of TB/FLU-04L in *Macaca fascicularis* (Fig. 4, S1, S2)

		Day 0		Days 1, 2	Days 2, 4, 6	Day 21		Day 42
Group	Number of animals	NW, Blood collection	1st Immunization TB/FLU-04L 0.5 ml 7.5 log$_{10}$ TCID$_{50}$/animal i/n	NW	NS	Blood collection (HAI, LA)	2nd Immunization TB/FLU-04L 0.5 ml 7.5 log$_{10}$ TCID$_{50}$/animal i/n	Blood collection (HAI, LA)
TB/FLU-04L	7	7/7	7/7	7/7	7/7	7/7	7/7	7/7

Seven adult male *Macaca fascicularis* were immunized twice with TB/FLU-04 (7.5 log$_{10}$TCID$_{50}$/animal), with a three-week interval between vaccinations. Blood samples for HAI assay and PBMCs were collected before each vaccination (day 0, day 21) and three weeks after the second vaccination (day 42). Body weight and temperature measurements were carried out before the first immunization and on days 2, 4, 8, 21, and 42.

Table S5. Experimental design: protective efficacy evaluation in mice (Fig 5)

		Day 0		Day 21	Day 42		5 weeks p.ch	20 weeks p.ch
Group	Number of animals	1st Immunization		Immunization	*M. tuberculosis* Erdman challenge		Euthanasia	
TB/FLU-04L	10	TB/FLU-04L 0.01 ml 6.0 log$_{10}$ TCID$_{50}$/animal i.n	10/10	10/10	0.01 ml 6.0 log$_{10}$ CFU/ animal i.v	10/10	5/10	5/10
BCG	10	BCG 0,01 ml 5.0 log$_{10}$ CFU/animal s.c	10/10	0/10		10/10	5/10	5/10
Non-vaccinated control	10	DPBS 0.01 ml i/n	10/10	10/10		10/10	5/10	3/10

Protective immunity against the intravenous challenge with virulent *M. tuberculosis* in mice following intranasal vaccination with TB/FLU-04L. C57BL/6 mice received saline i.n. (naive), or 1×10^5 CFU BCG s.c., or TB/FLU-04L i.n. twice with three-week intervals. Three weeks after the second vaccination, mice were challenged by an i.v. injection of 10^6 CFU of virulent *M. tuberculosis* Erdman strain. In 5 and 20 weeks after the challenge, five mice per group were sacrificed for histological examination and bacterial load determination.

Table S6. Experimental design: prime-boost protective efficacy in mice (Fig. 6, 7, 8)

		Day 0	Day 120	Day 141	Day 169 (4 weeks p.ch)			Up to Day 184
Group	Number of animals	Prime-Immunization BCG 0.01 ml 5.0 log_{10} CFU/animal s.c	Boost-Immunization TB/FLU-04L 0.01 ml 6.0 log_{10} $TCID_{50}$/animal i.n	*M. tuberculosis* Erdman challenge 0,01 ml 6.0 log_{10} CFU/ animal i.v	Euthanasia			Surviv al
					BL	Cyt, T_{reg}	H	
BCG	40	40/40	0/40	40/40	6/40	4/34	10/30	20/20
BCG+ TB/FLU-04L	40	40/40	40/40	40/40	6/40	4/34	10/30	20/20
Non-vaccinated control	40	0/40	0/40	40/40	6/40	4/34	10*/30	15/20**
Mock	4	0/4	0/4	0/4	0/4	4/4	-	-

The protective efficacy of the BCG prime with the TB/FLU-04L boost immunization against the i.v. *M. tuberculosis* challenge in C57BL/6 mice. C57BL/6 mice (40 animals per group) were immunized with a single s.c. dose of BCG (10^5 CFU; BCG group) or with one s.c. dose of BCG followed by one intranasal immunization with the influenza vector expressing ESAT-6 and Ag85A proteins (10^6 $TCID_{50,}$ BCG prime/TB/FLU-04 boost group) four months apart. The control group was immunized with PBS only at the time of the prime and boost immunizations (non-vaccinated control group). Three weeks after the boost immunization, mice were challenged intravenously (i.v.) with the virulent Erdman strain of *M. tuberculosis* (1 x 10^6 CFU). Four weeks after the challenge, the level of protection was measured by enumerating bacterial loads (CFU) in the lungs and spleens (6 animals per group) and evaluating gross pathological and histopathological changes in the lungs (10 animals per group). Cytokine secretion by spleen cells was measured at the time of sacrifice (4 mice per group). Survival of vaccinated and non-vaccinated C57BL/6 mice (15-20 animals per group) following the i.v. infection with *M. tuberculosis* Erdman was monitored over the 184-day observation period.

BL – bacterial loads, Cyt – cytokines, H – histopathology, p.ch. – post-challenge

**- one animal died before euthanasia (histological analysis was made for 9 animals in this group)*

***- 5 animals were sacrificed during the experiment to control the progression of tuberculosis*

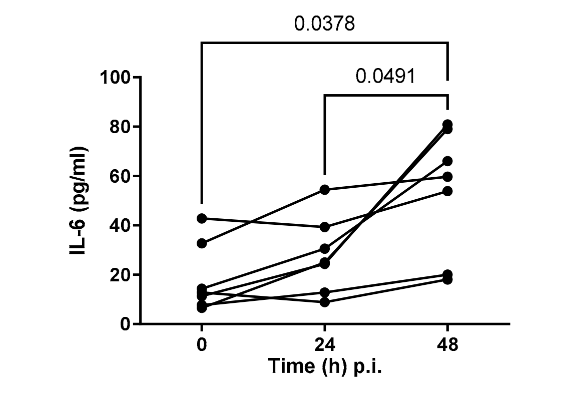

Animal №	Time (h) p.i.		
	0	24	48
**36112**	32.68	54.45	59.7
**37410**	11.31	24.36	80.86
**36955**	6.55	24.93	79.00
**38544**	42.82	39.32	53.89
**36580**	7.71	12.78	20.06
**37439**	14.31	30.58	66.07
**38649**	12.78	8.85	18.08

Figure S1. Local IL-6 response after the i.n. TB/FLU-04L vaccination. Seven adult male *Macaca fascicularis* were immunized with TB/FLU-04 (7.5 $log_{10}TCID_{50}$/animal) twice spaced three weeks apart. Nasal swabs were collected before the immunization and 24 and 48 hours after the first immunization. IL-6 levels were determined by using the BD OptEIA™ Monkey ELISA Set. The data were considered statistically significant when p<0.05 (*) in the one-way ANOVA followed by Tukey's multiple comparisons test.

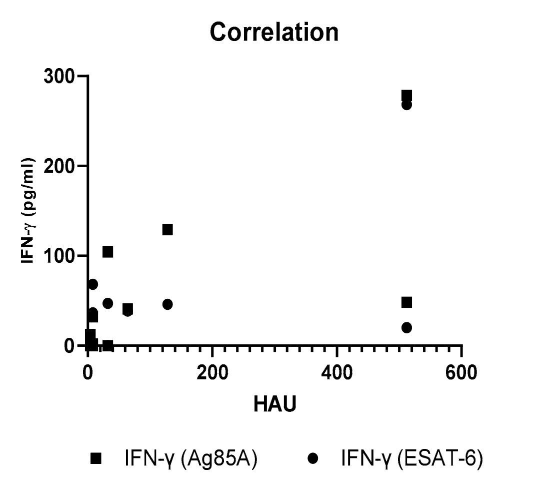

Pearson r	HAU vs. IFN-γ (ESAT-6)	HAUvs. IFN-γ (Ag85A)
r	0.6870	0.7275
95% confidence interval	0.3629 to 0.8628	0.4313 to 0.8822
R squared	0.4719	0.5293
P value		
P (two-tailed)	0.0006	0.0002
P value summary	***	***
Significant? (alpha = 0.05)	Yes	Yes
Number of XY Pairs	21	21

Figure S2. Pearson correlation analysis of antibody response to viral vector (HAI) and TB antigen-specific T-cell recall (IGRA).

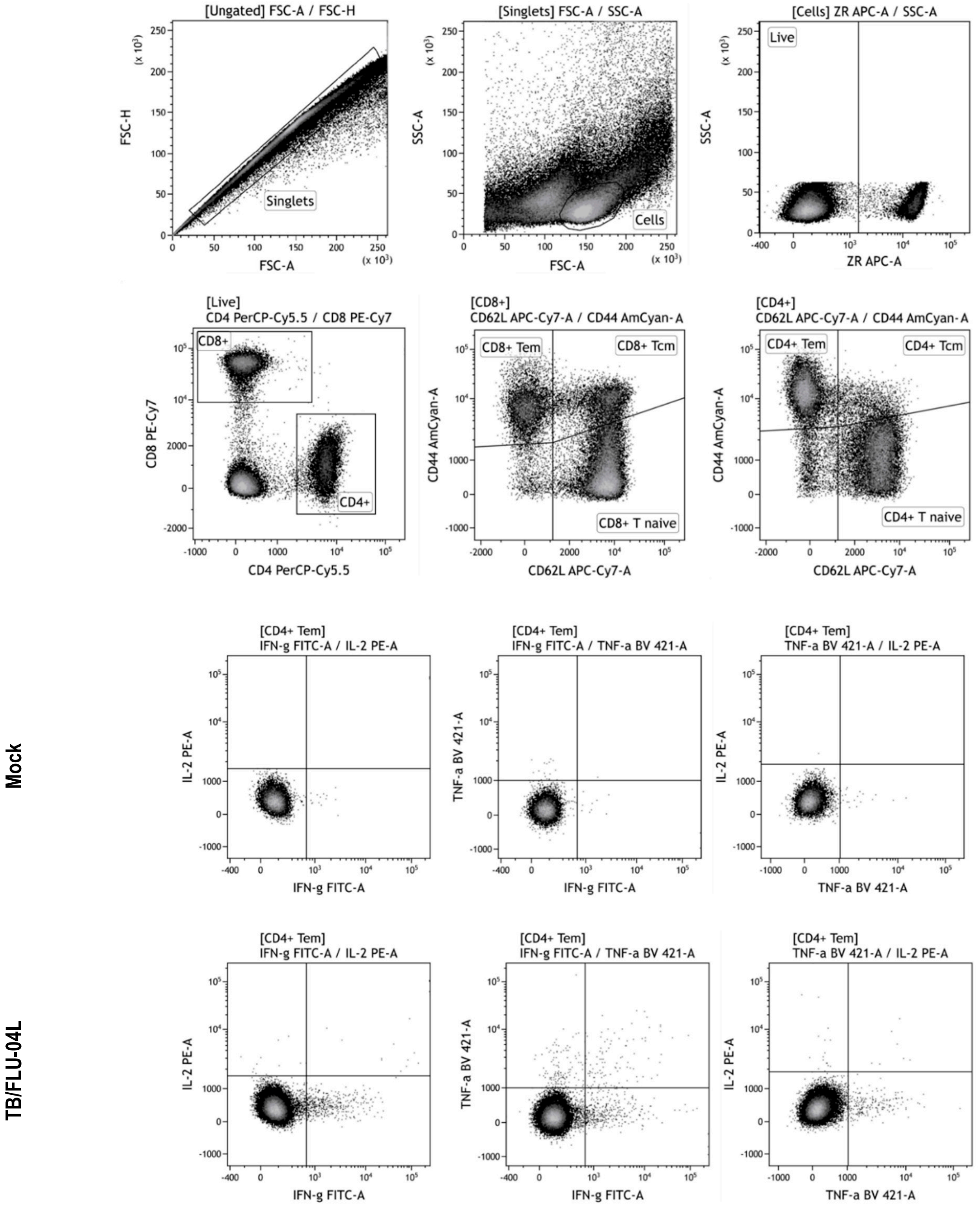

Figure S3. Gating strategy used to identify cytokine-producing Tem cells. Cell doublets were eliminated using FSC-A/FSC-H light scattering. The live single-cell population was determined based on the FSCA/SSC-A light scattering and binding of the Zombie Red dye. T helper cells were identified as CD4+, CTLs as CD8+. Using CD44 and CD62L markers, CD4+ and CD8+ cells were further subdivided into naïve (CD44-CD62L+), Tcm (CD44+CD62L+), and Tem (CD44+CD62L-) cells. IFN-γ, IL-2, and TNF-α responses were evaluated in Tem cells. Representative plots demonstrate Ag85A-induced cytokine production in cells derived from the lungs of control (Mock) and vaccinated (TB/FLU-04L) animals.

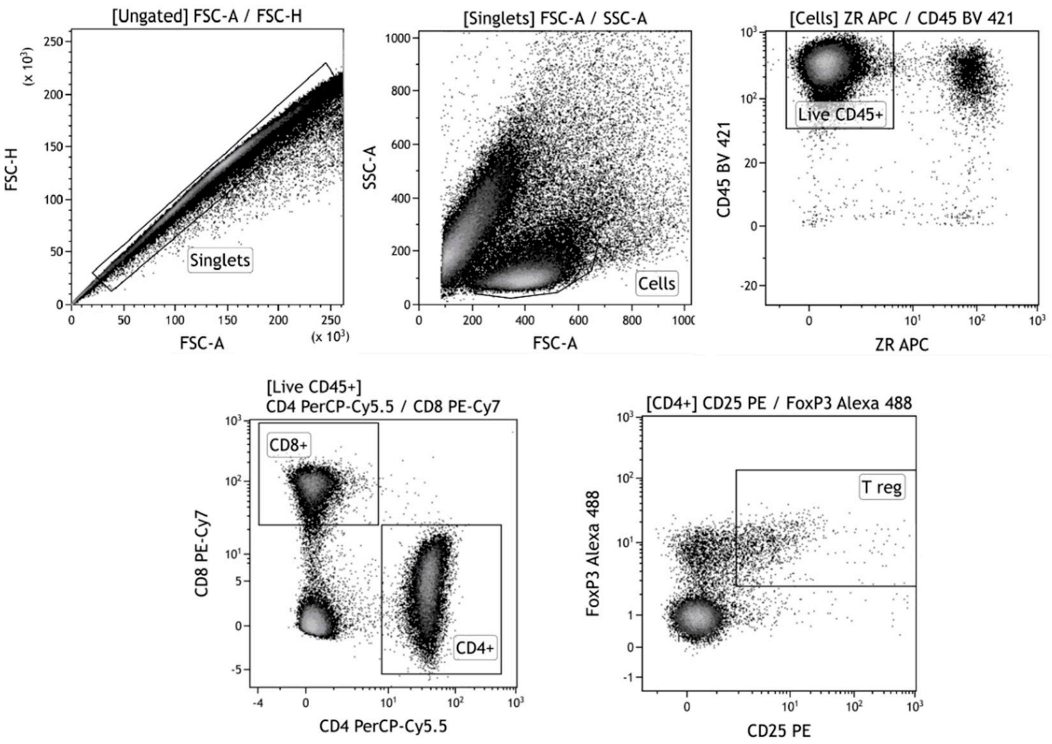

Figure S4. Gating strategy used to identify Treg cells. Cell doublets were eliminated using FSC-A/FSC-H light scattering. The live single-cell CD45+ population was identified based on the FSCA/SSC-A light scattering and binding of anti-CD45 antibodies and the Zombie Red dye. CD4+FoxP3+CD25+ population was determined as T reg cells.
